# Sodium samarium tetra­kis­(poly­phosphate), NaSm(PO_3_)_4_

**DOI:** 10.1107/S1600536810022543

**Published:** 2010-06-23

**Authors:** Dan Zhao, Lina Zhang, Feifei Li

**Affiliations:** a Department of Physics and Chemistry, Henan Polytechnic University, Jiaozuo, Henan 454000, People’s Republic of China

## Abstract

NaSm(PO_3_)_4_ has been prepared by solid state reactions. It belongs to type II of the structural family of *M* ^I^ *Ln* ^III^(PO_3_)_4_ compounds (*M* ^I^ = alkali metal and *Ln* ^III^ = rare earth metal) and is composed of _∞_(PO_3_)_*n*_]^*n*−^ polyphosphate chains with a repeating unit of four PO_4_ tetra­hedra. The chains extend parallel to [100] and share O atoms with irregular SmO_8_ polyhedra, forming a three-dimensional framework which delimits tunnels occupied by Na^+^ cations in a distorted octa­hedral environment.

## Related literature

The unit cell of NaSm(PO_3_)_4_, derived from X-ray powder data, was reported by Ferid *et al.* (1984 ▶). For classification of *M* ^I^ *Ln* ^III^(PO_3_)_4_ structures, see: Palkina *et al.* (1981 ▶); Durif (1995 ▶). Structures, properties and applications of other members of this family were discussed by Ettis *et al.* (2003 ▶); Parreu *et al.* (2007 ▶); Zhao *et al.* (2008 ▶, 2010 ▶); Zhu *et al.* (2009 ▶).

## Experimental

### 

#### Crystal data

NaSm(PO_3_)_4_
*M* *_r_* = 489.22
Monoclinic, 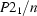

*a* = 7.1924 (13) Å
*b* = 13.091 (2) Å
*c* = 9.8480 (17) Å
β = 90.396 (10)°
*V* = 927.2 (3) Å^3^
*Z* = 4
Mo *K*α radiation
μ = 7.14 mm^−1^
*T* = 293 K
0.20 × 0.02 × 0.02 mm

#### Data collection

Rigaku Mercury70 CCD diffractometer
Absorption correction: multi-scan (*ABSCOR*; Higashi, 1995 ▶) *T*_min_ = 0.329, *T*_max_ = 0.870
7036 measured reflections
2111 independent reflections
1868 reflections with *I* > 2σ(*I*)
*R*_int_ = 0.041

#### Refinement

*R*[*F* ^2^ > 2σ(*F* ^2^)] = 0.037
*wR*(*F* ^2^) = 0.068
*S* = 1.15
2111 reflections
163 parameters
Δρ_max_ = 2.38 e Å^−3^
Δρ_min_ = −0.80 e Å^−3^

### 

Data collection: *CrystalClear* (Rigaku, 2004 ▶); cell refinement: *CrystalClear*; data reduction: *CrystalClear*; program(s) used to solve structure: *SHELXS97* (Sheldrick, 2008 ▶) and *PLATON* (Spek, 2009 ▶); program(s) used to refine structure: *SHELXL97* (Sheldrick, 2008 ▶); molecular graphics: *DIAMOND* (Brandenburg, 2004 ▶); software used to prepare material for publication: *SHELXTL* (Sheldrick, 2008 ▶).

## Supplementary Material

Crystal structure: contains datablocks I, global. DOI: 10.1107/S1600536810022543/wm2360sup1.cif

Structure factors: contains datablocks I. DOI: 10.1107/S1600536810022543/wm2360Isup2.hkl

Additional supplementary materials:  crystallographic information; 3D view; checkCIF report

## Figures and Tables

**Table 1:** Selected bond lengths (Å)

P1—O10	1.482 (4)
P1—O5	1.494 (4)
P1—O11	1.584 (4)
P1—O7	1.586 (4)
P2—O9	1.477 (4)
P2—O4	1.481 (4)
P2—O7^i^	1.574 (4)
P2—O6^ii^	1.598 (4)
P3—O1	1.482 (4)
P3—O12	1.486 (4)
P3—O6	1.591 (4)
P3—O3	1.596 (4)
P4—O8	1.479 (4)
P4—O2	1.487 (4)
P4—O11	1.594 (4)
P4—O3^i^	1.595 (4)

Symmetry codes: (i) 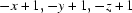; (ii) 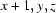.

**Fig. 1.**
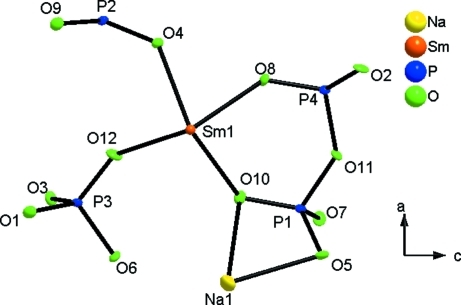
Asymmetric unit of the structure of NaSm(PO3)4 with the atom labelling scheme. The displacement ellipsoids are drawn at the 50% probability level.

**Fig. 2.**
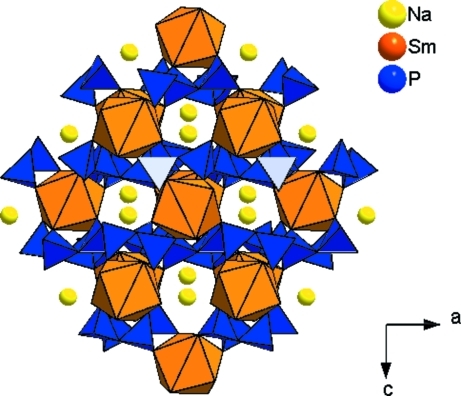
View of the crystal structure of NaSm(PO3)4 in a projection down [010].

## supplementary crystallographic information

### Comment

In recent years, alkali rare earth polyphosphates with general formula *M*^I^*Ln*^III^(PO_3_)_4_ (*M*^I^ = alkali metal, *Ln*^III^ = rare earth metal) have been studied mainly due to their rich structural chemistry and interesting physical and chemical properties, such as high luminescence efficiency (Durif, 1995). The nomenclature of *M*^I^*Ln*^III^(PO_3_)_4_ compounds has been proposed by Palkina *et al.* (1981) and is generally accepted today. Many compounds of this family have been reported, for example, Li*Ln*(PO_3_)_4_ (*Ln* =Y, Sm, Dy) (Zhao *et al.*, 2008, 2010), KGdP_4_O_12_ (Ettis *et al.*, 2003), KNd(PO_3_)_4_ (Parreu *et al.*, 2007) and CsEu(PO_3_)_4_ (Zhu *et al.*, 2009). For the compound NaSm(PO_3_)_4_ only unit-cell parameters have been reported on basis of refined X-ray powder diffraction data (Ferid *et al.*, 1984).

The structure of NaSm(PO_3_)_4_ is characterized by a three-dimensional framework built up of irregular SmO_8_ polyhedra linked with polyphosphate chains *via* Sm–O–P bridges, as show in Fig. 2. The undulated [(PO_3_)*_n_*]*^n^*^-^ chains have a repeating unit of four corner-sharing PO_4_ tetrahedra and extend parallel to the *a*-axis. Furthermore, the framework delimits tunnels in which the Na^+^ ions are located. They are 6-coordinated by O atoms with Na—O distances ranging from 2.386 (5)—2.741 (5) Å in a distorted octahedral arrangement.

### Experimental

The finely ground reagents Na_2_CO_3_, Sm_2_O_3_, and NH_4_H_2_PO_4_ were mixed in a molar ratio of Na: Sm: P = 7: 1: 10, placed in a Pt crucible, and heated at 673 K for 4 h. The mixture was re-ground and heated at 1173 K for 20 h, then cooled to 673 K at a rate of 4 K h^-1^, and finally quenched to room temperature. A few yellow crystals of the title compound with prismatic shape were obtained.

### Refinement

The highest peak in the difference electron density map is located 1.49 Å from atom Sm1 while the deepest hole is 1.98 Å from atom O8.

### Crystal data

**Table:**

NaSm(PO_3_)_4_	*F*(000) = 916
*M**_r_* = 489.22	*D*_x_ = 3.504 Mg m^−^^3^
Monoclinic, *P*2_1_/*n*	Mo *K*α radiation, λ = 0.71073 Å
Hall symbol: -P 2yn	Cell parameters from 2180 reflections
*a* = 7.1924 (13) Å	θ = 2.1–27.5°
*b* = 13.091 (2) Å	µ = 7.14 mm^−^^1^
*c* = 9.8480 (17) Å	*T* = 293 K
β = 90.396 (10)°	Prism, yellow
*V* = 927.2 (3) Å^3^	0.20 × 0.02 × 0.02 mm
*Z* = 4	

### Data collection

**Table:**

Rigaku Mercury70 CCD diffractometer	2111 independent reflections
Radiation source: fine-focus sealed tube	1868 reflections with *I* > 2σ(*I*)
Rigaku Graphite Monochromator DIFFRACTOMETER TYPE	*R*_int_ = 0.041
Detector resolution: 14.6306 pixels mm^-1^	θ_max_ = 27.5°, θ_min_ = 2.6°
ω scans	*h* = −9→9
Absorption correction: multi-scan (*ABSCOR*; Higashi, 1995)	*k* = −15→17
*T*_min_ = 0.329, *T*_max_ = 0.870	*l* = −12→12
7036 measured reflections	

### Refinement

**Table:**

Refinement on *F*^2^	0 restraints
Least-squares matrix: full	Primary atom site location: structure-invariant direct methods
*R*[*F*^2^ > 2σ(*F*^2^)] = 0.037	Secondary atom site location: difference Fourier map
*wR*(*F*^2^) = 0.068	*w* = 1/[σ^2^(*F*_o_^2^) + (0.0639*P*)^2^] where *P* = (*F*_o_^2^ + 2*F*_c_^2^)/3
*S* = 1.15	(Δ/σ)_max_ < 0.001
2111 reflections	Δρ_max_ = 2.38 e Å^−^^3^
163 parameters	Δρ_min_ = −0.80 e Å^−^^3^

### Special details

**Table:**

Geometry. All e.s.d.'s (except the e.s.d. in the dihedral angle between two l.s. planes) are estimated using the full covariance matrix. The cell e.s.d.'s are taken into account individually in the estimation of e.s.d.'s in distances, angles and torsion angles; correlations between e.s.d.'s in cell parameters are only used when they are defined by crystal symmetry. An approximate (isotropic) treatment of cell e.s.d.'s is used for estimating e.s.d.'s involving l.s. planes.
Refinement. Refinement of *F*^2^ against ALL reflections. The weighted *R*-factor *wR* and goodness of fit *S* are based on *F*^2^, conventional *R*-factors *R* are based on *F*, with *F* set to zero for negative *F*^2^. The threshold expression of *F*^2^ > σ(*F*^2^) is used only for calculating *R*-factors(gt) *etc*. and is not relevant to the choice of reflections for refinement. *R*-factors based on *F*^2^ are statistically about twice as large as those based on *F*, and *R*- factors based on ALL data will be even larger.

### Fractional atomic coordinates and isotropic or equivalent isotropic displacement parameters (Å^2^)

**Table:**

	*x*	*y*	*z*	*U*_iso_*/*U*_eq_	
Na1	−0.0002 (4)	0.7220 (2)	0.5648 (3)	0.0210 (6)	
Sm1	0.51300 (4)	0.71807 (2)	0.47634 (3)	0.00716 (10)	
P1	0.2493 (2)	0.60065 (11)	0.74392 (15)	0.0064 (3)	
P2	0.8781 (2)	0.61480 (12)	0.26404 (15)	0.0063 (3)	
P3	0.2690 (2)	0.59026 (12)	0.19783 (15)	0.0065 (3)	
P4	0.6463 (2)	0.62775 (12)	0.80511 (15)	0.0065 (3)	
O1	0.2374 (6)	0.6610 (3)	0.0824 (4)	0.0117 (9)	
O2	0.7180 (5)	0.7105 (3)	0.8947 (4)	0.0099 (9)	
O3	0.2829 (6)	0.4784 (3)	0.1343 (4)	0.0091 (9)	
O4	0.8029 (6)	0.6454 (3)	0.3976 (4)	0.0095 (9)	
O5	0.0936 (6)	0.6652 (3)	0.7951 (4)	0.0103 (9)	
O6	0.0886 (6)	0.5804 (3)	0.2895 (4)	0.0105 (9)	
O7	0.2159 (6)	0.4860 (3)	0.7898 (4)	0.0098 (9)	
O8	0.6799 (6)	0.6328 (3)	0.6572 (4)	0.0100 (9)	
O9	0.8672 (6)	0.6896 (3)	0.1519 (4)	0.0105 (9)	
O10	0.2860 (6)	0.6073 (3)	0.5963 (4)	0.0102 (9)	
O11	0.4284 (6)	0.6243 (3)	0.8331 (4)	0.0082 (9)	
O12	0.4293 (6)	0.6102 (3)	0.2901 (4)	0.0135 (9)	

### Atomic displacement parameters (Å^2^)

**Table:**

	*U*^11^	*U*^22^	*U*^33^	*U*^12^	*U*^13^	*U*^23^
Na1	0.0172 (14)	0.0309 (16)	0.0149 (12)	0.0063 (13)	−0.0028 (11)	0.0029 (12)
Sm1	0.00640 (16)	0.00857 (15)	0.00651 (15)	−0.00002 (13)	0.00033 (11)	−0.00033 (13)
P1	0.0049 (7)	0.0065 (7)	0.0078 (7)	−0.0001 (6)	−0.0001 (6)	−0.0006 (6)
P2	0.0055 (7)	0.0053 (7)	0.0082 (7)	0.0002 (6)	0.0012 (6)	0.0002 (6)
P3	0.0046 (7)	0.0064 (7)	0.0084 (7)	0.0013 (6)	0.0000 (6)	−0.0001 (6)
P4	0.0053 (7)	0.0059 (7)	0.0084 (7)	−0.0007 (6)	0.0001 (6)	−0.0007 (6)
O1	0.010 (2)	0.013 (2)	0.012 (2)	0.0027 (18)	0.0012 (18)	0.0029 (18)
O2	0.005 (2)	0.009 (2)	0.016 (2)	−0.0013 (17)	−0.0032 (17)	−0.0044 (18)
O3	0.014 (2)	0.005 (2)	0.009 (2)	0.0016 (17)	−0.0019 (17)	−0.0014 (17)
O4	0.007 (2)	0.012 (2)	0.009 (2)	0.0009 (17)	0.0012 (17)	−0.0007 (17)
O5	0.005 (2)	0.013 (2)	0.013 (2)	0.0014 (17)	0.0020 (17)	−0.0007 (18)
O6	0.008 (2)	0.015 (2)	0.008 (2)	−0.0019 (18)	0.0034 (17)	0.0022 (18)
O7	0.014 (2)	0.005 (2)	0.011 (2)	−0.0042 (17)	−0.0001 (18)	−0.0013 (16)
O8	0.010 (2)	0.013 (2)	0.0064 (19)	0.0015 (18)	0.0028 (17)	0.0026 (17)
O9	0.011 (2)	0.008 (2)	0.012 (2)	0.0023 (17)	0.0018 (18)	0.0028 (17)
O10	0.012 (2)	0.010 (2)	0.009 (2)	−0.0015 (18)	0.0019 (18)	−0.0004 (17)
O11	0.007 (2)	0.010 (2)	0.0076 (19)	−0.0012 (17)	−0.0028 (17)	−0.0021 (16)
O12	0.008 (2)	0.015 (2)	0.017 (2)	−0.0020 (18)	−0.0044 (18)	−0.0047 (18)

### Geometric parameters (Å, °)

**Table:**

Na1—O4^i^	2.386 (5)	P2—O6^vii^	1.598 (4)
Na1—O1^ii^	2.438 (5)	P3—O1	1.482 (4)
Na1—O2^iii^	2.468 (5)	P3—O12	1.486 (4)
Na1—O5	2.475 (5)	P3—O6	1.591 (4)
Na1—O10	2.565 (5)	P3—O3	1.596 (4)
Na1—O8^i^	2.741 (5)	P4—O8	1.479 (4)
Na1—O9^ii^	3.005 (5)	P4—O2	1.487 (4)
Sm1—O9^ii^	2.362 (4)	P4—O11	1.594 (4)
Sm1—O12	2.389 (4)	P4—O3^vi^	1.595 (4)
Sm1—O8	2.415 (4)	O1—Na1^iv^	2.438 (5)
Sm1—O5^iv^	2.423 (4)	O1—Sm1^iii^	2.486 (4)
Sm1—O4	2.424 (4)	O2—Sm1^v^	2.449 (4)
Sm1—O2^iii^	2.449 (4)	O2—Na1^v^	2.468 (5)
Sm1—O1^v^	2.486 (4)	O3—P4^vi^	1.595 (4)
Sm1—O10	2.488 (4)	O4—Na1^vii^	2.386 (5)
P1—O10	1.482 (4)	O5—Sm1^ii^	2.423 (4)
P1—O5	1.494 (4)	O6—P2^i^	1.598 (4)
P1—O11	1.584 (4)	O7—P2^vi^	1.574 (4)
P1—O7	1.586 (4)	O8—Na1^vii^	2.741 (5)
P2—O9	1.477 (4)	O9—Sm1^iv^	2.362 (4)
P2—O4	1.481 (4)	O9—Na1^iv^	3.005 (5)
P2—O7^vi^	1.574 (4)		
			
O4^i^—Na1—O1^ii^	81.77 (16)	O10—P1—O5	115.9 (2)
O4^i^—Na1—O2^iii^	93.40 (16)	O10—P1—O11	112.5 (2)
O1^ii^—Na1—O2^iii^	108.62 (17)	O5—P1—O11	108.1 (2)
O4^i^—Na1—O5	131.55 (19)	O10—P1—O7	111.3 (2)
O1^ii^—Na1—O5	109.24 (16)	O5—P1—O7	108.9 (2)
O2^iii^—Na1—O5	123.97 (17)	O11—P1—O7	98.7 (2)
O4^i^—Na1—O10	108.05 (17)	O9—P2—O4	117.8 (2)
O1^ii^—Na1—O10	168.60 (18)	O9—P2—O7^vi^	106.5 (2)
O2^iii^—Na1—O10	77.17 (15)	O4—P2—O7^vi^	111.6 (2)
O5—Na1—O10	60.02 (14)	O9—P2—O6^vii^	110.5 (2)
O4^i^—Na1—O8^i^	63.56 (14)	O4—P2—O6^vii^	106.8 (2)
O1^ii^—Na1—O8^i^	65.92 (15)	O7^vi^—P2—O6^vii^	102.8 (2)
O2^iii^—Na1—O8^i^	156.55 (17)	O1—P3—O12	118.2 (3)
O5—Na1—O8^i^	78.01 (15)	O1—P3—O6	111.4 (2)
O10—Na1—O8^i^	112.69 (17)	O12—P3—O6	107.4 (2)
O4^i^—Na1—O9^ii^	151.14 (16)	O1—P3—O3	106.4 (2)
O1^ii^—Na1—O9^ii^	114.66 (17)	O12—P3—O3	110.5 (2)
O2^iii^—Na1—O9^ii^	59.56 (14)	O6—P3—O3	101.6 (2)
O5—Na1—O9^ii^	67.70 (14)	O8—P4—O2	119.5 (3)
O10—Na1—O9^ii^	59.17 (13)	O8—P4—O11	109.8 (2)
O8^i^—Na1—O9^ii^	143.89 (15)	O2—P4—O11	104.8 (2)
P1—Na1—O9^ii^	60.64 (10)	O8—P4—O3^vi^	110.7 (2)
O9^ii^—Sm1—O12	138.95 (15)	O2—P4—O3^vi^	107.7 (2)
O9^ii^—Sm1—O8	85.27 (14)	O11—P4—O3^vi^	102.9 (2)
O12—Sm1—O8	114.50 (15)	P3—O1—Sm1^iii^	144.7 (2)
O9^ii^—Sm1—O5^iv^	109.05 (14)	Na1^iv^—O1—Sm1^iii^	94.05 (16)
O12—Sm1—O5^iv^	82.37 (15)	P4—O2—Sm1^v^	140.3 (2)
O8—Sm1—O5^iv^	135.61 (14)	P4—O2—Na1^v^	116.4 (2)
O9^ii^—Sm1—O4	145.34 (14)	Sm1^v^—O2—Na1^v^	101.18 (16)
O12—Sm1—O4	74.63 (14)	P4^vi^—O3—P3	132.3 (3)
O8—Sm1—O4	68.31 (13)	P2—O4—Na1^vii^	120.5 (2)
O5^iv^—Sm1—O4	78.51 (14)	P2—O4—Sm1	135.3 (2)
O9^ii^—Sm1—O2^iii^	69.92 (14)	Na1^vii^—O4—Sm1	97.01 (16)
O12—Sm1—O2^iii^	76.16 (14)	P1—O5—Sm1^ii^	142.1 (2)
O8—Sm1—O2^iii^	147.54 (13)	P1—O5—Na1	93.5 (2)
O5^iv^—Sm1—O2^iii^	74.25 (14)	Sm1^ii^—O5—Na1	114.88 (18)
O4—Sm1—O2^iii^	142.17 (13)	P3—O6—P2^i^	131.6 (3)
O9^ii^—Sm1—O1^v^	69.91 (14)	P2^vi^—O7—P1	139.8 (3)
O12—Sm1—O1^v^	149.17 (14)	P4—O8—Sm1	131.5 (2)
O8—Sm1—O1^v^	70.50 (14)	P4—O8—Na1^vii^	119.3 (2)
O5^iv^—Sm1—O1^v^	75.54 (14)	Sm1—O8—Na1^vii^	88.43 (14)
O4—Sm1—O1^v^	80.05 (14)	P2—O9—Sm1^iv^	149.9 (3)
O2^iii^—Sm1—O1^v^	116.94 (14)	P2—O9—Na1^iv^	120.7 (2)
O9^ii^—Sm1—O10	69.79 (14)	Sm1^iv^—O9—Na1^iv^	89.30 (14)
O12—Sm1—O10	81.81 (14)	P1—O10—Sm1	128.5 (2)
O8—Sm1—O10	72.84 (14)	P1—O10—Na1	90.3 (2)
O5^iv^—Sm1—O10	151.45 (14)	Sm1—O10—Na1	97.49 (15)
O4—Sm1—O10	119.46 (14)	P1—O11—P4	135.0 (3)
O2^iii^—Sm1—O10	78.97 (14)	P3—O12—Sm1	139.8 (3)
O1^v^—Sm1—O10	126.71 (13)		

Symmetry codes: (i) *x*−1, *y*, *z*; (ii) *x*−1/2, −*y*+3/2, *z*+1/2; (iii) *x*−1/2, −*y*+3/2, *z*−1/2; (iv) *x*+1/2, −*y*+3/2, *z*−1/2; (v) *x*+1/2, −*y*+3/2, *z*+1/2; (vi) −*x*+1, −*y*+1, −*z*+1; (vii) *x*+1, *y*, *z*.
